# Epigenetic and Genomic Hallmarks of PARP-Inhibitor Resistance in Ovarian Cancer Patients

**DOI:** 10.3390/genes15060750

**Published:** 2024-06-07

**Authors:** Tugce Senturk Kirmizitas, Caroline van den Berg, Ruben Boers, Jean Helmijr, Stavros Makrodimitris, Hamit Harun Dag, Marijn Kerkhofs, Corine Beaufort, Jaco Kraan, Wilfred F. J. van IJcken, Joost Gribnau, Pakriti Garkhail, Gatske Nieuwenhuyzen-de Boer, Eva-Maria Roes, Heleen van Beekhuizen, Tuba Gunel, Saskia Wilting, John Martens, Maurice Jansen, Ingrid Boere

**Affiliations:** 1 University Medical Center Rotterdam, Department of Medical Oncology, Erasmus MC, 3015 GD Rotterdam, The Netherlands; t.senturkkirmizitas@erasmusmc.nl (T.S.K.); j.helmijr@erasmusmc.nl (J.H.); s.makrodimitris@erasmusmc.nl (S.M.); h.dag@erasmusmc.nl (H.H.D.); m.kerkhofs@erasmusmc.nl (M.K.); c.beaufort@erasmusmc.nl (C.B.); j.kraan@erasmusmc.nl (J.K.); s.wilting@erasmusmc.nl (S.W.); j.martens@erasmusmc.nl (J.M.); i.boere@erasmusmc.nl (I.B.); 2 Institute of Graduate Studies in Sciences, Istanbul University, Istanbul 34116, Turkey; 3 University Medical Center Rotterdam, Department of Gynecological Oncology, Erasmus MC, 3015 GD Rotterdam, The Netherlands; c.vandenberg@erasmusmc.nl (C.v.d.B.); p.garkhail@erasmusmc.nl (P.G.); g.nieuwenhuyzen-deboer@erasmusmc.nl (G.N.-d.B.); e.roes@erasmusmc.nl (E.-M.R.); h.vanbeekhuizen@erasmusmc.nl (H.v.B.); 4 University Medical Center Rotterdam, Department of Developmental Biology, Erasmus MC, 3015 GD Rotterdam, The Netherlands; r.g.boers@erasmusmc.nl (R.B.); j.gribnau@erasmusmc.nl (J.G.); 5 University Medical Center Rotterdam, Center of Biomics, Erasmus MC, 3015 GD Rotterdam, The Netherlands; w.vanijcken@erasmusmc.nl; 6 Department of Molecular Biology & Genetics, Istanbul University, Istanbul 34134, Turkey; gunel@istanbul.edu.tr

**Keywords:** ovarian cancer, cell-free DNA, mFast-SeqS, MeD-seq, shWGS, exome-seq, PARP-inhibitors, epigenetic and genomic hallmarks

## Abstract

Background: Patients with advanced-stage epithelial ovarian cancer (EOC) receive treatment with a poly-ADP ribose-polymerase (PARP) inhibitor (PARPi) as maintenance therapy after surgery and chemotherapy. Unfortunately, many patients experience disease progression because of acquired therapy resistance. This study aims to characterize epigenetic and genomic changes in cell-free DNA (cfDNA) associated with PARPi resistance. Materials and Methods: Blood was taken from 31 EOC patients receiving PARPi therapy before treatment and at disease progression during/after treatment. Resistance was defined as disease progression within 6 months after starting PARPi and was seen in fifteen patients, while sixteen patients responded for 6 to 42 months. Blood cfDNA was evaluated via Modified Fast Aneuploidy Screening Test-Sequencing System (mFast-SeqS to detect aneuploidy, via Methylated DNA Sequencing (MeD-seq) to find differentially methylated regions (DMRs), and via shallow whole-genome and -exome sequencing (shWGS, exome-seq) to define tumor fractions and mutational signatures. Results: Aneuploid cfDNA was undetectable pre-treatment but observed in six patients post-treatment, in five resistant and one responding patient. Post-treatment ichorCNA analyses demonstrated in shWGS and exome-seq higher median tumor fractions in resistant (7% and 9%) than in sensitive patients (7% and 5%). SigMiner analyses detected predominantly mutational signatures linked to mismatch repair and chemotherapy. DeSeq2 analyses of MeD-seq data revealed three methylation signatures and more tumor-specific DMRs in resistant than in responding patients in both pre- and post-treatment samples (274 vs. 30 DMRs, 190 vs. 57 DMRs, Χ^2^-test *p* < 0.001). Conclusion: Our genome-wide Next-Generation Sequencing (NGS) analyses in PARPi-resistant patients identified epigenetic differences in blood before treatment, whereas genomic alterations were more frequently observed after progression. The epigenetic differences at baseline are especially interesting for further exploration as putative predictive biomarkers for PARPi resistance.

## 1. Introduction 

High-grade serous ovarian cancer (HGSOC) is the most common (75%) histological type found in advanced-stage epithelial ovarian cancer (EOC) [1,2]. The different histological subtypes of ovarian cancer, such as HGSOC, low-grade serous, endometrioid and clear cell are characterized by differences in their molecular profile. HGSOC is characterized by mutations in *TP53* in >90% of cases and by mutations in *BRCA1* or *BRCA2* in around 20% of cases. Other genomic alterations include copy number variations. Most patients are treated with chemotherapy and debulking surgery as the standard of care, but many patients develop disease recurrence and eventually die from the disease. Clearly, there is a need for improvement. 

One of the most successful strategies has been the use of poly-ADP ribose-polymerase (PARP) inhibitors (PARPi), especially for patients with a somatic or germline *BRCA* mutation. Since the approval of the first PARPi in 2014, initially approved as a maintenance therapy after the response to chemotherapy for patients with a *BRCA* mutation, these drugs have expanded to encompass first-line maintenance treatment in EOC patients with or without a *BRCA* mutation. In parallel, major efforts are ongoing to select which patients will respond best to PARPi therapy.

A few studies have investigated blood cell-free DNA (cfDNA) from ovarian cancer patients and linked cfDNA alterations to PARPi resistance. Most of these studies observed genomic changes [3,4,5,6,7,8,9,10]. These studies showed *BRCA1/2* reversion mutations in cfDNA from PARPi-resistant patients [5,8,9]. Moreover, an *MRE11* p.K446R mutation was also frequently found in cfDNA from patients resistant to Olaparib [6], and one study showed in cell line models that this mutation resulted in the reduced accumulation of cellular DNA damage [3]. Only one study analyzed cfDNA methylation in PARPi-treated patients and showed that *HOXA9* cfDNA methylation was related to poor outcomes in patients receiving PARPi [11]. 

Our hypothesis is that cfDNA epigenetic and genomic markers can help to predict resistance to PARPi in patients with a first recurrence of EOC. This study aims to simultaneously evaluate cfDNA genomic and methylation alterations in EOC patients and relate these changes to PARPi resistance. Therefore, we evaluated blood cfDNA before and after the PARPi maintenance therapy of 31 HGSOC patients with and without therapy resistance using several genome-wide Next-Generation Sequencing (NGS) assays. 

## 2. Materials and Methods

### 2.1. Study Design and Participants

**Figure 1:**
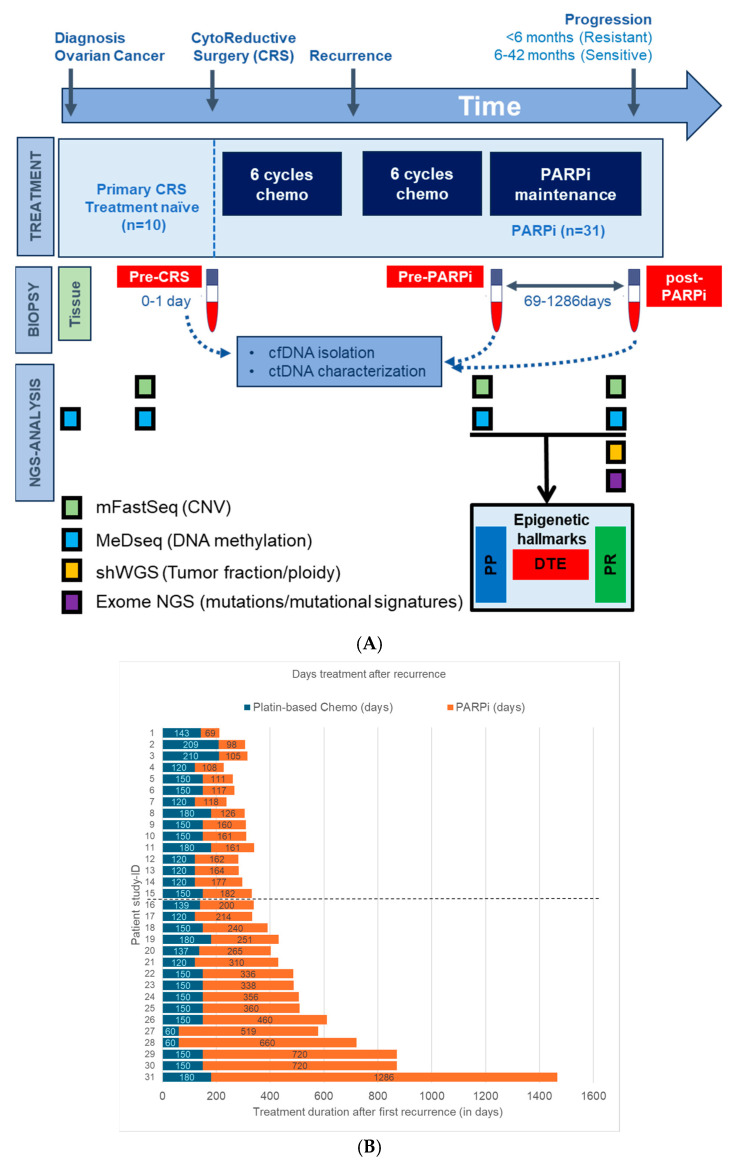
Study design, patient cancer treatment, Next-Generation Sequencing (NGS) overview. (**A**) This study analyzed blood from 31 High-grade serous ovarian cancer (HGSOC) patients who received poly-ADP ribose-polymerase inhibitor (PARPi) maintenance therapy, blood from 8 healthy blood donors (HBDs), and tumor tissue with matched treatment-naïve blood from 10 HGSOC patients. Genome-wide NGS analyses were performed on cancer and normal blood samples and in part also on tumor tissue. dynamic tumor evolution (DTE), PARPi predictive (PP), and PARPi response (PR) indicate the timepoints where the epigenetic hallmarks were defined. (**B**) Plot showing the chemotherapy and PARPi therapy duration after first recurrence for all HGSOC patients. Patients above the dotted horizontal line are defined as PARPi-resistant, while those below the dotted line are defined as PARPi-sensitive.

Blood samples for cfDNA analysis of EOC patients were obtained from the Liquid Biopsy Bank (LBB), a local biobank of the Erasmus MC Medical University Center, Rotterdam, the Netherlands. Anonymous female healthy blood donors (HBD) were obtained from the Sanquin Blood Bank and patient recruitment was performed at the Cancer Institute of the Erasmus Medical Center (Rotterdam, The Netherlands) between 2018 and 2021, with written informed consent obtained from all individuals prior to sample collection. Ethical approval was granted by the Erasmus MC Medical Ethical Committee (MEC-2017-238, 8 May 2017). In total, 49 participants were selected for this study: 41 EOC patients diagnosed with first recurrent advanced-stage HGSOC (Figure 1A) and 8 female HBDs. Ten patients with tumor tissue and treatment naïve blood (TN-blood) at diagnosis and eight HBDs were used as a reference set for cfDNA profiling. Blood specimens were also collected from 31 HGSOC patients treated with PARPi maintenance therapy (olaparib or niraparib). Blood in this subset was taken at two distinct time points: before treatment initiation and at disease progression during PARPi treatment. Three patients did not progress during maintenance therapy; blood from these patients was taken after stopping PARPi therapy. Fifteen patients had disease progression within 6 months of treatment initiation and were grouped as PARPi-resistant. Conversely, 16 patients responded for more than 6 months (range: 6 to 42 months) to PARPi therapy and were classified as PARPi-sensitive. 

All blood was collected in CellSave preservative tubes and, within 4 days, processed into plasma after centrifugation at 1700× *g* and at 12,000× *g* for 10 min each and stored at −80 °C. Plasma cell-free DNA was isolated using the QIAamp Circulating Nucleic Acid kit (Qiagen, Venlo, The Netherlands). The cfDNA was quantified by means of the Qubit 4.0 fluorometer using the dsDNA HS Assay kit (Thermo Fisher, Waltham, MA, USA) and fragment sizes were measured using the QIAxcel system (Qiagen). Genomic DNA from formalin-fixed paraffin-embedded (FFPE) tumor tissue from the 10 EOC patients of the reference set was isolated with the AllPrep DNA/RNA FFPE Kit (Qiagen). All cfDNA and genomic DNA were then stored at −20 °C before further analyses. Next, a comprehensive multi-omics analysis was performed using the modified fast aneuploidy screening test-sequencing system, methylated DNA sequencing and shallow whole-genome and -exome sequencing (Figure 1).

### 2.2. Modified Fast Aneuploidy Screening Test-Sequencing System (mFast-SeqS)

For the mFast-SeqS analysis, focusing on aneuploidy detection, we used blood samples from all EOC patients before and after PARPi treatment. mFast-SeqS offers a low-resolution method to detect tumor-derived aneuploidy in cfDNA of cancer patients. The mFast-SeqS protocol was implemented and modified according to Belic et al. [12] and Verschoor et al. [13]. Briefly, Line-1 (L1) amplicon libraries were prepared from minute cfDNA input amounts (1 ng). The PCR reaction mixture consisted of Phusion HF Buffer, Phusion Hot Start II Polymerase, target-specific L1 primer, and dNTPs. Subsequently, PCR products underwent purification using AMPure Beads, followed by a second PCR step where sequencing adaptors and sample-specific indexes were added. The sample setup and cycling conditions remained consistent with the first PCR, albeit with increased cycle numbers. Libraries from 46 samples were combined in equimolar proportions and sequenced on the MiSEQ platform (Illumina, San Diego, CA, USA), yielding a minimum of 90 thousand single-end reads of 150 base pairs.

### 2.3. Methylated DNA Sequencing (MeD-Seq)

For the MeD-seq analysis, all 49 samples were used, encompassing both reference sets and blood samples collected before and after PARPi treatment. The MeD-seq assay was applied to 10 ng of cell-free DNA and genomic DNA extracted from all plasma and tissue samples, respectively, as previously described for methylation analysis [14]. This method employed the methylation-dependent enzyme called LpnPI to target methylated or hydroxy methylated cytosines in specific sequences, generating 32 base pair fragment snippets. After digestion, sequencing adaptors are ligated to the DNA fragments and the library was amplified. Multiplexed samples were sequenced on the HiSeq 2500 platform system (Illumina), yielding a minimum of 20 million 50 base pair single-end reads. 

### 2.4. Shallow Whole Genome and Exome Sequencing (shWGS/Exome-Seq)

For shWGS and exome-seq, we analyzed blood from 8 HBDs and blood collected from 30 of the 31 HGSOC patients, exclusively post-PARPi treatment. For one patient, insufficient cfDNA amounts were available, and these samples were therefore excluded for the shWGS/exome-seq analyses. The WGS library preparation was conducted using 20 ng cfDNA as the input and the Twist Library Preparation Kit with the UMI Adapter System according to the manufacturer’s instructions. By leveraging the Twist UMI Adapter System, libraries were prepared with UMI tags and quantified by means of qPCR using Equinox Library Amp Mix, allowing for the accurate quantification and analysis of sequencing data. Parts of the generated WGS-amplified libraries were used for exome capture (36.5 Mb targets) using probes. WGS and Exome-Seq multiplex libraries were sequenced on the NovaSeq platform (Illumina), yielding a minimum of 11.4 and 2.5 million paired-end reads of 150 bases and a median depth coverage of 3.7 and 161 reads, respectively. 

### 2.5. Data Processing

Primer sequences were removed from the mFast-SeqS sequencing outcomes using trimmomatic before aligning them to the human reference genome hg38 using Burrows–Wheeler alignment (v0.7.17). Reads with mapping qualities of less than 15 were discarded, and the remaining reads were used to calculate the total read count per chromosomal arm. Due to insufficient LINE-1 elements, the short arms of the acrocentric chromosomes 13p, 14p, 15p, and 22p were excluded from further analysis. For MeD-Seq, data processing involved trimming and filtering based on LpnPI restriction site occurrence. The processed reads were aligned with the genome, and count scores were assigned to individual LpnPI sites. Measures included the total read count, the fraction passing the LpnPI filter, and the fraction of duplicate reads. CpG site count scores were aggregated into 2-kilobase regions surrounding transcription start sites (TSS). Subsequently, read counts per TSS from 60 thousand TSSs were evaluated using DeSeq2. The DeSeq2 tool is typically employed for differential expression analysis of RNA-Seq count data using a robust statistical framework including normalization and variance estimation. DeSeq2 has also been applied by others to determine differential DNA methylation [15,16]. Since our Med-Seq also results in count-based methylation data, we used DeSeq2 to identify differentially methylated regions (DMRs) with high sensitivity and specificity. Normalization and square root transformation were applied on read counts after DeSeq2 for downstream analyses and plotting. Only regions on autosomal chromosomes were considered and regions without counts in more than 75% of samples were excluded from further analysis. For shWGS and exome sequencing, FastQC was used to assess the quality of the FastQ files. Trimmomatic (v0.32) was used to trim Illumina adapter sequences. Sequence reads were aligned with human genome hg38 reference version using the variant caller developed by HMF (https://github.com/hartwigmedical/pipeline, accessed on 16 October 2023).

### 2.6. Data Interpretation

#### 2.6.1. mFast-SeqS Genome-Wide Aneuploidy Z-Score (GWZ)

From mFasSeqS data, a GWZ-score for aneuploidy was computed per chromosomal arm, representing the deviation from a reference panel of healthy diploid subjects. This score was determined by subtracting the mean and dividing by the standard deviation of normalized read-counts for the corresponding chromosome arm, allowing for the evaluation of over- and under-representation. Then, the GWZ-scores for each chromosome arm were squared and then summed to compute a GWZ for aneuploidy for each patient. Different GWZ aneuploidy thresholds were evaluated to classify aneuploid cfDNA. Following the threshold outlined by Belic et al. [12], samples with a GWZ of 5 or higher had a high tumor load (>10%) and are appropriate for subsequent shWGS and/or exome-Seq analyses.

#### 2.6.2. Methylation Analysis: Selection and Subclassification of DMRs 

To comprehensively analyze methylation profiles across diverse sources and time points, we used the statistical R package DeSeq2. More specifically, to find disease- and tumor-related DMRs, we compared MeD-seq data with DeSeq2 from all cancer blood and tissue samples as specific subsets with the HBD reference set. DMRs were established for each disease subset and only those DMRs which had a *p*-value below 0.05 when adjusted for multiple testing (FDR/Benjamini–Hochberg correction) were selected. These significant DMRs were used to define epigenetic PARPi hallmarks. Finally, DMRs from the pre-PARPi subset were evaluated by means of Cox proportional hazard regression analyses to identify DMRs that were significantly (*p* < 0.05) related to time to treatment failure (TTF) for PARPi therapy. 

#### 2.6.3. Comprehensive Pathway Analyses

The three different epigenetic PARPi therapy hallmarks were evaluated in Metascape using custom settings to find enriched pathways in each DMR signature. Our analysis included Gene Ontology (GO) biological processes, Kyoto Encyclopedia of Genes and Genomes (KEGG) pathways, immunologic signatures, oncogenic signatures, transcription factor targets, and Transcriptional Regulatory Relationships Unraveled by Sentence-based Text mining (TRRUST) databases. All statistically enriched terms were found based on custom settings. Accumulative hypergeometric *p*-values and enrichment factors were then computed and used for filtering the significant terms. Remaining significant terms underwent hierarchical clustering into a tree based on Kappa-statistical similarities among their gene memberships. A kappa score threshold of 0.3 was applied to partition the tree into distinct term clusters. In addition, DMRs from the epigenetic hallmarks were linked to the COSMIC database of tumor driver genes (TDGs) and the homologous recombination deficiency (HRD) gene list defined in a pan-cancer landscape analyses for homologous recombination [17]. The identified DMRs were also linked to the cancer hallmark pathways (CHPs) to assess whether epigenetic PARPi hallmarks were associated with cancer-specific pathways.

#### 2.6.4. shWGS and Exome-Seq: Tumor Fraction and Ploidy Estimation, CNV and Mutation Analysis

Tumor fractions and ploidy levels were estimated from shWGS and exome sequencing data using the ichorCNA tool from the Broad Institute (Cambridge, MA, USA) with the default settings. Mutational signatures were found using the SigMiner tool (version 2.3.0) from the comprehensive R Archive Network. The mutational signatures were found in exome-seq data. Briefly, input variants were filtered for their variant allele frequencies (VAFs). To enrich tumor-specific mutations and remove putative germ-line variants as much as possible, we selected only variants detected in at least 10 reads and with VAFs below 45% or between 55% and 70%. Subsequently, variants detected in the HBDs were also removed and the remaining variants were used to detect Single-base substitution (SBS) and Insertion-Deletion (ID) signatures described in the COSMIC database (cancer.sanger.ac.uk/signatures). Briefly, after filtering out variants detected in healthy individuals, the remaining variants were subjected to mutational signature analysis using SigMiner. This computational tool enabled the identification of specific mutational patterns known as signatures, as described in the COSMIC database. To quantify the influence of each mutational signature, we calculated the fraction of mutations attributed to each signature relative to the total number of mutations observed within a sample. This approach allowed us to discern the relative impact of different mutational processes on the genomic landscape of our study cohort.

#### 2.6.5. Statistical Analysis and Plotting

Results were plotted using online tools (https://www.bioinformatics.com.cn/en, accessed on 20 March 2024; https://jvenn.toulouse.inrae.fr/app/example.html, accessed on 15 March 2024; http://www.heatmapper.ca/, accessed on 25 March 2024). Statistical analyses were performed using Stata statistical package release 17 (STATA Corp., College Station, TX, USA) and Simple Interactive Statistical Analysis (SISA) statistics (https://www.quantitativeskills.com/sisa/index.htm, accessed on 29 March 2024), employing Pearson’s correlation, Student’s *t*-test, the chi square test, and Fisher’s exact test. In addition, a Cox proportional hazard regression analysis was performed using TTF. TTF was defined as the time from the start of treatment to disease progression or stopping PARPi treatment. Differences with *p*-values below 0.05 were considered statistically significant.

## 3. Results

The main goal of this study was to characterize the genomic and epigenetic hallmarks of PARPi therapy resistance as well as disease progression in HGSOC patients. The design of this study is shown in Figure 1. Briefly, genome-wide NGS was performed on 31 HGSOC patients, namely on blood plasma cfDNA from PARPi-resistant (*n* = 15) and -sensitive patients (*n* = 16) (Figure 1A). All patients received platin-based chemotherapy prior to PARPi therapy (Figure 1B). Eleven patients displayed a *BRCA1/2* wildtype in the germline and tumor DNA, equally distributed amongst the two PARPi response groups (Supplemental Table S1). HBDs and the tissue and treatment-naïve blood of ten HGSOC-patients were also used and analyzed as reference sets. To detect genomic alterations, pre- and post-treatment blood was evaluated by means of mFastSeq, while post-treatment blood was also analyzed by means of shWGS and exome-seq. MeD-seq was performed on all samples from HGSOC patients and HBDs to determine DMRs. These DMRs were established in pre- and post-treatment blood relative to HBDs and were used to define three different epigenetic PARPi hallmarks. Unique DMRs from pre-and post-treatment blood representing methylation changes during disease progression and PARPi treatment were defined as dynamic tumor evolution hallmarks (DTE). Unique DMRs from PARPi-resistant and -sensitive patients in pre-treatment samples taken before disease progression and PARPi-treatment were defined as PARPi predictive hallmarks (PP). Unique DMRs from PARPi-resistant and-sensitive patients in post-treatment samples taken after disease progression and/or PARPi-treatment were defined as PARPi response hallmarks (PR). The study is described in more detail below.

### 3.1. General Patient and Blood Characteristics 

The clinicopathological characteristics for the 31 HGSOC patients were compared between PARPi-resistant (*n* = 15) and -sensitive patients (*n* = 16) (Table 1). None of the parameters were significantly different between the two patient subsets, except for the PARPi response, which was used to categorize patients into the two PARP response groups. In addition, cfDNA yields per mL of plasma were compared between blood collection timepoints (before and after treatment) and patient subsets. No significant differences were seen in cfDNA yields between pre- and post-treatment blood (median: 7.5 ng/mL and 8.9 ng/mL; Student *t*-test, *p* = 0.45). The cfDNA yields between resistant and sensitive patients were comparable in pre-treatment blood (median: 7.3 and 7.6 ng/mL) and increased in post-treatment blood (median: 11.0 and 8.0 ng/mL), but were not different at both timepoints between the two patient subsets (Student *t*-test, *p* = 0.41 and *p* = 0.18), respectively.

### 3.2. Genomic Hallmarks in Blood cfDNA from PARPi-Resistant and -Sensitive Patients

**Figure 2:**
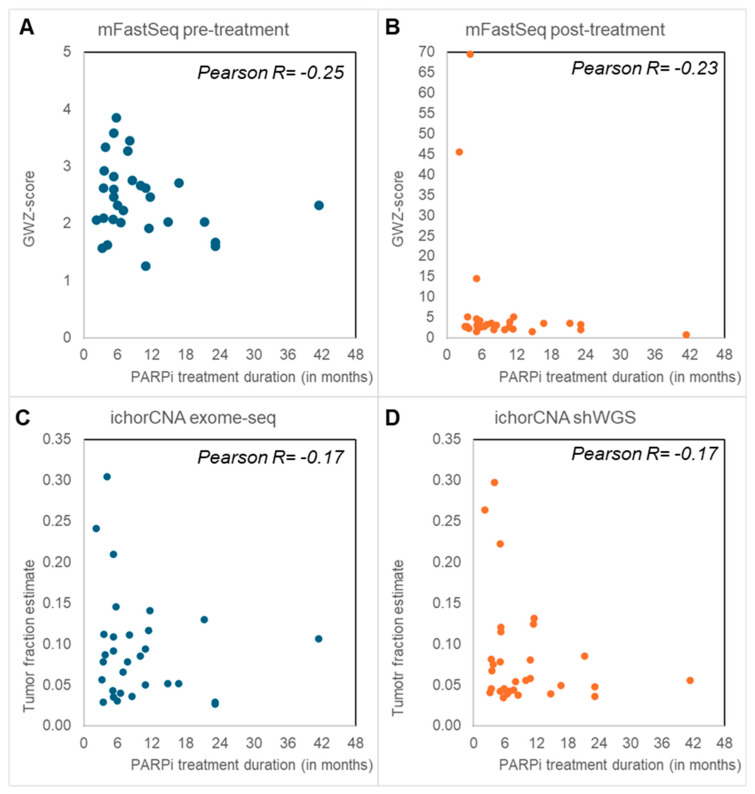
Genomic hallmarks: Aneuploid cell free DNA (cfDNA) and tumor fraction. Blood taken before the start of poly-ADP ribose-polymerase inhibitor (PARPi) (**A**) and after PARPi treatment (**B**–**D**) was evaluated by means of Modified Fast Aneuploidy Screening Test-Sequencing System (mFast-SeqS) (**A**,**B**) and shallow whole-genome and -exome sequencing (shWGS, exome-seq) (**C**,**D**). The Next-Generation sequencing (NGS) outcomes had all an inverse correlation with PARPi treatment duration. (**A**,**B**) mFast-SeqS analysis was performed to determine genome-wide Z (GWZ)-scores as a measure for aneuploid cfDNA. Pre-treatment blood (**A**) only had GWZ-scores below 5. GWZ-scores of 5 or higher with high aneuploid cfDNA levels were only seen in post-treatment blood (**B**), especially in patients resistant to PARPi treatment who had disease progression within 6 months. (**C**,**D**) IchorCNA analyses were performed to define tumor fractions from exome-seq (**C**) and shWGS (**D**). Tumor fractions above 10% were predominantly seen in patients with disease progression within 6 months after PARPi therapy.

The mFast-Seq analyses to detect circulating tumor DNA (ctDNA) via aneuploidy only revealed GWZ-scores below 5 in all pre-treatment samples, irrespective of PARPi response (Figure 2). Blood with a GWZ-score above 5 has been reported to have a high ctDNA load [12], and thus these findings indicate no or low levels of ctDNA in the pre-treatment samples. In contrast, six post-treatment samples displayed high GWZ-scores above 5, all except one from PARPi-resistant patients. IchorCNA analyses of shWGS and Exome-seq data from post-treatment blood found tumor fractions above 10% in seven and eleven samples, respectively (Figure 2). Again, high tumor fractions were more often seen in PARPi-resistant (median tumor fractions of 9% and 7%) than -sensitive patients (median tumor fractions 7% and 5%) and confirmed most of the post-treatment mFast-Seq findings (Supplemental Figure S1). 

**Figure 3:**
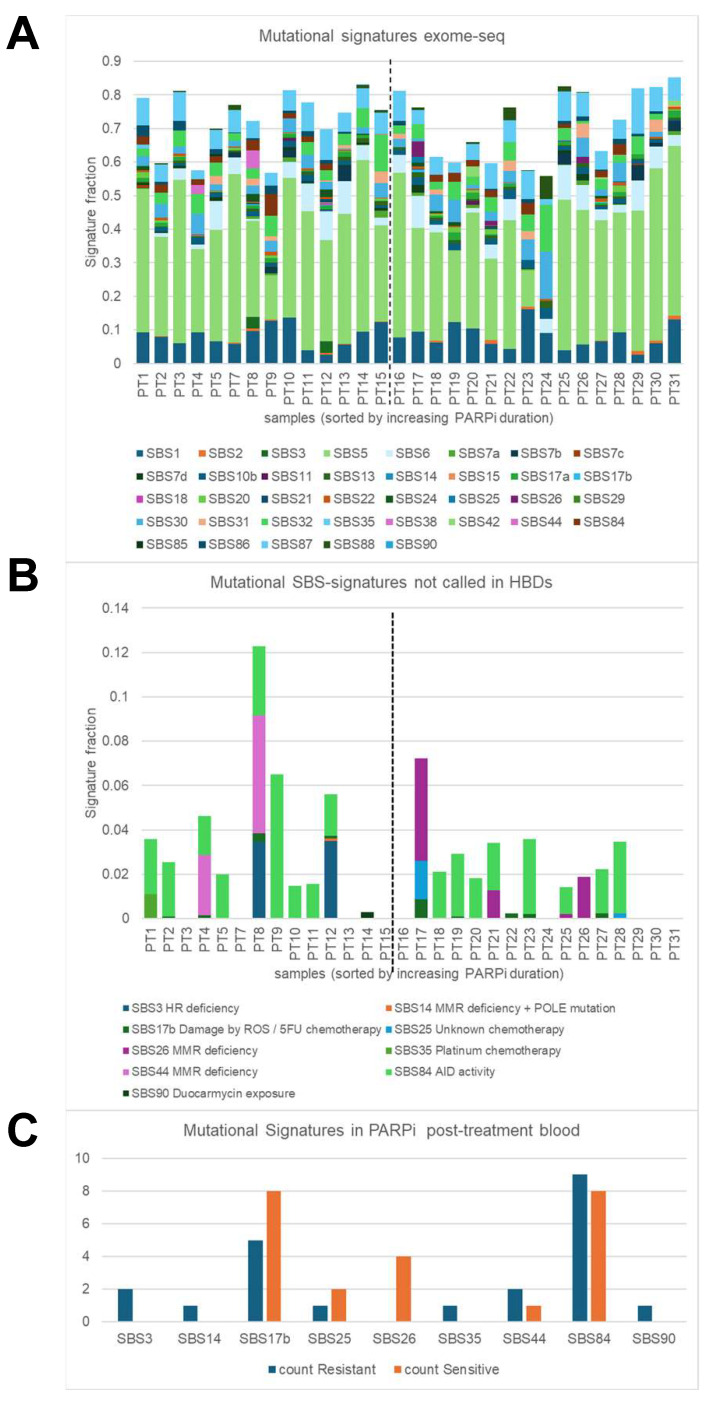
Genomic hallmarks: mutational signatures. Single-base substitution (SBS) mutational signatures were characterized in blood taken at disease progression after poly-ADP ribose-polymerase inhibitor (PARPi) from 30 High-grade serous ovarian cancer (HGSOC) patients using the SigMiner tool and exome-seq data. (**A**) Only 37 SBS signatures with known mutational processes are evaluated and presented in this plot. The vertical dashed line indicates the border between resistant patients (samples on the left) and sensitive patients (samples on the right). A total signature fraction below 70% was seen in 3 resistant samples (21%) and in 7 sensitive samples (44%). The signature fraction is determined by dividing the number of mutations assigned to a particular mutational signature by the number of all observed mutations within a sample. (**B**) Plot showing 9 annotated SBS signatures which were present in cancer blood but not in healthy blood donors (HBD). (**C**) Plot presenting the incidence of the 9 SBS signatures in resistant and sensitive patients. SBS26 was present in four sensitive patients but not in resistant patients (*p* = 0.04).

Next, mutational signatures defined by SigMiner were evaluated in post-treatment exome-seq samples. Analyses were focused on 52 mutational signatures (43 SBSs and 9 IDs) with a known mutational process (Figure 3A) and identified 9 SBS signatures and one ID signature in ovarian PARPi samples which were not seen in HBDs (Figure 3B,C). The observed mutational processes were linked to HR and MMR deficiency (SBS3, SBS14, SBS26, SBS44), chemotherapy (SBS17B (5-FU-related), SBS25, SBS35 (platinum)), AID activity (SBS84), duocarmycin exposure (SBS90), and colibactin exposure (ID18). The identification of SBS25, SBS84, SBS90, and ID18 was unexpected, while all other SBS signatures can be linked to the systemic therapy that the ovarian cancer patients in our cohort received. All SBS signatures except for SBS26 were seen in at least one PARPi-resistant patient, while only five (SBS17B, SBS25, SBS26, SBS44, and SBS84) were observed in PARPi-sensitive patients. SBS26 was seen in four PARPi-sensitive samples but not in PARPi-resistant samples (*p* = 0.04), and alongside SBS14 and SBS44 is one of the seven signatures associated with defective DNA mismatch repair. The SBS26-positive patients displayed a *BRCA1/2* wildtype for the germline and tumor (*n* = 2), a germline wild type (*n* = 1), or had a germline *BRCA2* mutation (*n* = 1) and were treated with niraparib (*n* = 3) or olaparib (*n* = 1). The SBS3 signature, which was strongly associated with germline and somatic *BRCA1/2* mutations and *BRCA1* methylation in other studies, was seen in our study in only two samples; however, both were obtained from *BRCA1/2* germline and tumor wild-type patients. The identification of chemotherapy-linked SBS35 makes sense since the patients in this cohort received platinum-based chemotherapy before PARPi maintenance therapy. 

To summarize the genomic hallmarks, aneuploid cfDNA and high tumor fractions were predominantly seen in post-treatment blood from PARPi-resistant patients, while the SBS26 signature for defective DNA mismatch repair was observed in PARPi-sensitive patients.

### 3.3. Epigenetic Hallmarks

#### 3.3.1. Detection of Ovarian Cancer DMRs

**Figure 4:**
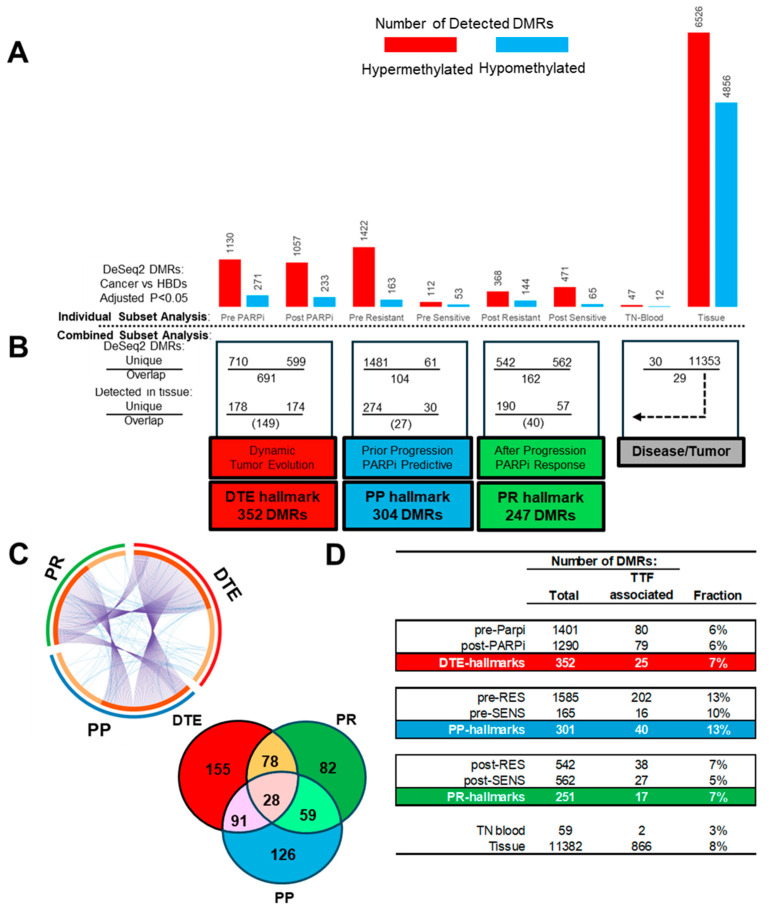
Epigenetic hallmarks: Differential methylated regions (DMRs) at transcription start sites (TSS) were determined with DeSeq2 by comparing cancer samples with healthy blood donors (HBDs). Cancer samples included poly-ADP ribose-polymerase inhibitor (PARPi) blood subsets and a treatment-naïve (TN) blood and matched tumor tissue subset. (**A**) Counts of hyper- and hypomethylated DMRs for the different cancer blood and tissue sample subsets. (**B**) Sample subsets were combined and compared within subclasses, and only subset unique DMRs which were also seen in tissue were used to define three different epigenetic PARPi hallmarks. (**C**) Circular and Venn plot showing the unique and overlapping DMRs between the epigenetic hallmarks. (**D**) DMRs from the pre-PARPi subset were evaluated via Cox proportional hazard logistic regression to find DMRs significantly associated with time to treatment failure (TTF) for PARPi therapy in all subsets and hallmark subclasses.

To find the epigenetic hallmarks for EOC and PARPi resistance, we first defined the TSS regions that are differentially methylated between disease and healthy samples using DeSeq2. For this, generated MeD-seq data from the samples included in the study were used to find DMRs with an adjusted *p*-value < 0.05 across comparisons between cancer sample subsets and the HBDs as the reference subset (Figure 4A). These analyses revealed that each cancer subset showed unique DNA methylation alterations, characterized by varying proportions of hypo- and hypermethylated DMRs. The highest numbers of DMRs were found in tissue and PARPi-resistant blood, while treatment-naïve blood had the lowest number of DMRs. All subsets more frequently had hyper- than hypomethylated DMRs. 

#### 3.3.2. Defining PARPi-Related Epigenetic Hallmarks

The above detected disease-related DMRs were compared within four cancer subclasses to define PARPi-specific epigenetic hallmarks (Figure 4B). The first analysis compared the pre- and post-PARPi treatment subsets to find methylation changes over time during PARPi treatment and disease progression and was defined as DTE. This comparison resulted in 710 and 599 DMRs only found at the start or after treatment, respectively, while 694 DMRs were seen in both subsets. The second and third subclass compared resistant and sensitive patients to define DMRs related to PARPi therapy response in pre- and post-treatment subsets, respectively. The first PARPi-response subclass (called PP) used pre-treatment subsets, prior to disease progression and treatment outcome, thus selecting predictive DMRs. The second PARPi response subclass (called PR) used post-treatment subsets and detected DMRs at disease progression (or at treatment termination) after PARPi therapy. This latter comparison provides information on tumor biology beyond therapy failure. The comparisons in the pre- and post-treatment subclasses resulted in 1481 and 542 DMRs which were only seen in resistant patients, while 61 and 562 DMRs were exclusively seen in sensitive patients. The overlap within these two subclasses resulted in 104 and 162 DMRs. Finally, the fourth subclass was defined as disease/tumor and compared the DMRs found in treatment-naïve blood at diagnosis with the DMRs detected in matched tumor tissue. This resulted in 30 and 11,353 unique DMRs for blood and tissue, respectively, and 29 matching DMRs found in both. This finding suggests that only half of the disease-related DMRs in the blood originates from tumor cells. The DMRs from the tissue subset were, for this reason, used to select tumor-related DMRs in the PAPRi blood subsets and subclasses (Figure 4B). 

From all the tumor-related unique DMRs, three different epigenetic PARPi therapy hallmarks were defined: the DTE hallmark, composed of 352 DMRs, and the PP and PR hallmarks, composed of 304 and 247 DMRs, respectively. The PP and PR hallmarks had more tumor-specific DMRs in resistant than in responding patients in both pre- and post-treatment samples (274 vs. 30 DMRs, 190 vs. 57 DMRs, Χ^2^-test *p* < 0.001) (Figure 4B). The three epigenetic PARPi hallmarks had overlapping DMRs in addition to hallmark-specific DMRs for DTE (155 DMRs), PP (126 DMRs), and PR (82 DMRs) (Figure 4C). All DMRs from the pre-PARPi subset were also analyzed with Cox proportional hazard regression analyses to find DMR profiles associated with PARPi TTF (*p* < 0.05) as a continuous variable instead of the categorical variables, resistant versus sensitive, applied in DeSeq2 (Figure 4D). The number of TTF-associated DMRs was determined in all subsets and epigenetic hallmarks. As expected, the PP hallmarks had more TTF-related DMRs and a higher fraction (40 DMRs, 13%) than the DTE and PR hallmarks (25 and 17 DMRs, 6% and 7%) (Χ^2^-tests *p* < 0.03).

### 3.4. Exploring Epigenetic Hallmarks and DMR Linkages to Known Tumor Driver and HRD Genes

**Figure 5:**
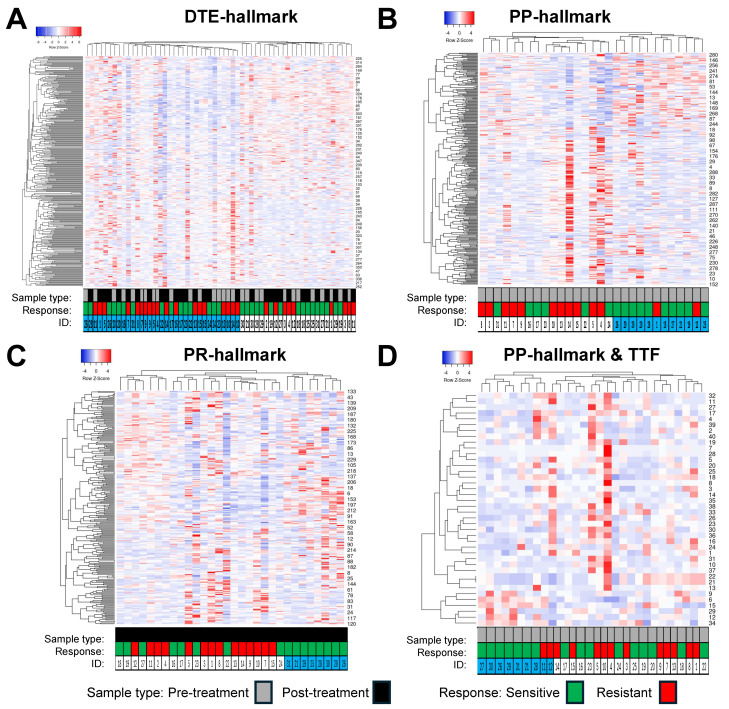
Hierarchical clustering of epigenetic poly-ADP ribose-polymerase inhibitor (PARPi) hallmark differentially Methylated Regions (DMRs). DMRs and samples were evaluated by Heatmapper using the average linkage as the clustering method and Spearman rank correlation as the distance measurement method. Columns represent samples with IDs below the heatmap, rows represent DMRs with their labels on the right indicated by numbers. (**A**) This cluster heatmap shows the DMRs from the dynamic tumor evolution (DTE) hallmark. All pre- and post-treatment samples were clustered. The left cluster arm had 37 samples with 19 pre- and 18 post-treatment samples, while the right cluster arm had 22 samples containing 10 pre- and 12 post-treatment samples. In addition, 19 and 18 samples in the left cluster and 9 and 13 samples in the right cluster were from resistant and sensitive patients, respectively. (**B**,**D**) Samples from 14 resistant and 16 sensitive patients were grouped by different hallmark DMRs. (**B**) This cluster heatmap shows the PARPi predictive (PP) hallmark DMRs in pre-treatment samples. Clustering resulted in two major groups of samples, with those from resistant patients mainly seen in the left cluster (12/14 patients), while samples from sensitive patients are found in the right cluster (10/16 patients). (**C**) This heatmap shows PARPi resistance (PR) hallmark DMRs in post-treatment samples. Clustering resulted in three groups of samples: the left cluster with 7 samples, the middle cluster with 15 samples, and the right cluster with 8 samples. The samples from resistant patients are seen in the left cluster (4 samples) and in the middle cluster (10 samples), while samples from sensitive patients are found in all three clusters, with the right cluster containing samples from sensitive patients only. (**D**) Heatmap of 40 DMRs derived from the PP hallmarks which were significantly related to time to treatment failure (TTF) after PARPi therapy using the (same) samples from the prePARPi subset presented in (**B**).

The expression profiles of the different epigenetic PARPi hallmarks were evaluated by means of hierarchical cluster analyses using Heatmapper and cluster metrics average linkage and Spearman rank correlation (Figure 5). The DMRs from the DTE and PP hallmarks (Figure 5A,B) resulted each in two distinct clusters of samples. The DTE hallmark DMRs did not display a specific enrichment of samples within each cluster, either for pre- versus post-treatment or for sensitive versus resistant. In contrast, the PP hallmark DMRs for pre-treatment samples displayed predominant clusters of samples from resistant (left cluster) and sensitive (right cluster) patients. The DMRs from the PR hallmarks (Figure 5C) resulted in three clusters of post-treatment samples. Samples from resistant patients were grouped into two distinct clusters (left and middle cluster, 4 and 10 samples, respectively), while samples from sensitive patients were found in one distinct cluster (right cluster, 8 samples), while the remaining samples were divided between the other two clusters. Finally, 40 DMRs from the PP hallmarks which were significantly associated with TTF were used to cluster the pre-treatment samples. This also resulted in three clusters, with the left and right clusters mainly containing samples from sensitive (left cluster: seven out of nine samples) and resistant (right cluster: five out of nine samples) patients, respectively, while the middle cluster contained the remaining samples from each group of patients. The epigenetic PARPi hallmark DMRs were also linked to published gene lists of 722 tumor driver (TD) genes and 58 HRD genes to see whether these hallmarks contained already-known cancer- and PARPi-relevant genes. This analysis identified three TD genes that were seen in the DTE hallmarks (*SOX2*, *POU2AF1*, and *PTK6*), while two TD genes were found in the PP hallmarks (*VHL*, *JAK3*) and in the PR hallmarks (*EZH2*, *SOX2*). Only one HRD gene (*RAD51C*) was detected in the DTE hallmark, while none were detected in the other two epigenetic hallmarks.

### 3.5. Functional Insights from DMR Signatures by Pathway Analysis

**Figure 6:**
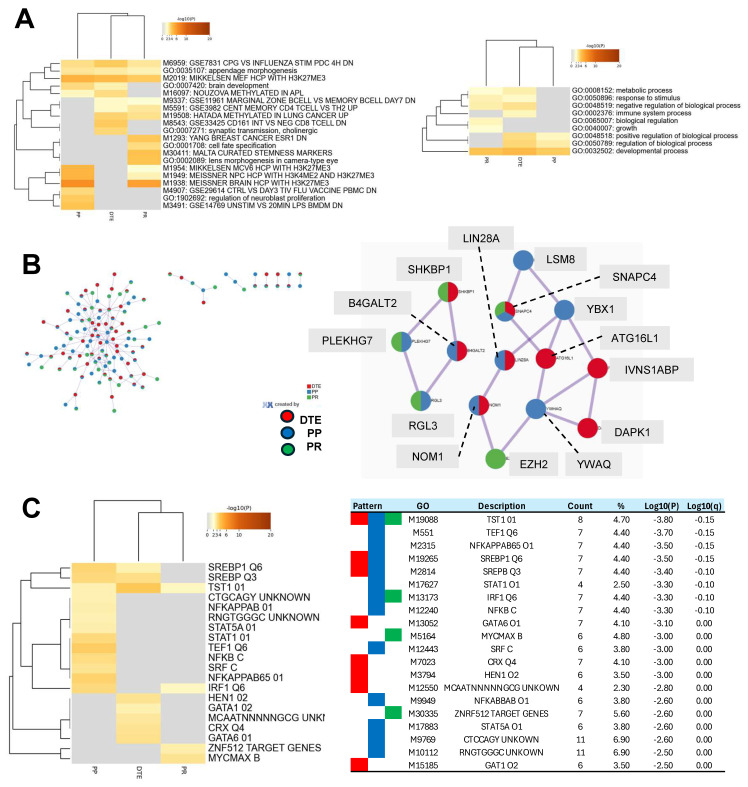
Comprehensive pathway analyses of epigenetic poly-ADP ribose-polymerase inhibitor (PARPi) hallmark differentially methylated regions (DMRs)**.** The DMRs from the epigenetic PARPi hallmarks were linked to genes and analyzed by Metascape using custom settings for a functional representation of gene signatures. Metascape evaluated 213, 194, and 155 genes from the dynamic tumor evolution (DTE), PARPi predictive (PP), and PARPi response (PR) hallmarks, respectively. (**A**) The cluster heatmap shows the enriched functional categories (left figure) and Gene Ontology (GO) processes (right figure) for all three epigenetic hallmarks. Examples of unique categories and processes were M8543 and GO:0002376 for the DTE hallmark, M3491 for the PP hallmark, and M1293 and GO:0065007 for the PR hallmark, while M2019 and GO:0032502 were seen in all hallmarks. (**B**) This figure presents all protein–protein interactions (left figure) and the two most significant protein interaction nodes (right figure). The first node contained the following hallmark unique proteins: *ATG16L1*, *IVNS1ABP*, *DAPK1* (from DTE), *LSM8*, *YWHAQ*, *YBX1* (from PP), and *EZH2* (from PR hallmark). (**C**) The cluster heatmap shows the enriched transcription factor targets for each of the epigenetic hallmarks. The table in the right figure presents details, including GO-IDs describing the targets.

Subsequently, pathway analysis was conducted in Metascape using custom settings to detect enriched functional categories, processes, protein–protein interactions, and transcription factor targets within the three epigenetic PARPi hallmarks. Metascape could not link all DMRs to genes, and, therefore only used 213, 194, and 155 unique genes as the input from the DTE, PP and PR hallmark DMRs (Figure 6).

Publicly available databases were explored for functional categories and processes and enriched entities were defined and compared between the epigenetic hallmarks. The top 20 most significant enriched categories are presented in Figure 6A. DTE was enriched for 10 functional entities, PP for 11 entities, and PR for 13 entities. Unique functional categories were observed for DTE (two entities), for PP (three entities), and for PR (four entities). Four enriched entities were related to H3K27me3, and were especially seen in PP and PR. On the other hand, three entities were linked to immune cells (M9337, M5591, and M8543) and were observed in DTE and PR. Next, nine Gene Ontology processes were enriched, with the immune system process only seen in DTE and biological regulation and growth only seen in PR. The PP hallmarks had three processes which were all also seen in DTE. Protein–protein interaction analyses identified in the most significant interaction node three proteins unique for each of the DTE and PP hallmarks and one protein for the PR hallmarks (Figure 6B). Finally, enrichment analyses of transcription factor targets demonstrated the largest number of targets in the PP hallmarks (13 targets), while DTE and PR had only eight and four targets (Figure 4). The *TST1* target (M19088) was seen in all epigenetic hallmarks and contains the motif NNKGAATTAVAVTDN within 4 kb around the transcription starting site of the *POU3F1*, *STAT,* and *NFKB* targets, which were enriched in the PP hallmark, while *GATA* targets were seen in the DTE hallmark, and *MYC* and *ZNF* targets were observed in the PR hallmark. All of these findings indicate that the three epigenetic PARPi hallmarks have subtle differences in their functional and biological processes, in their protein–protein interactions, and in their transcription factor targets.

**Figure 7:**
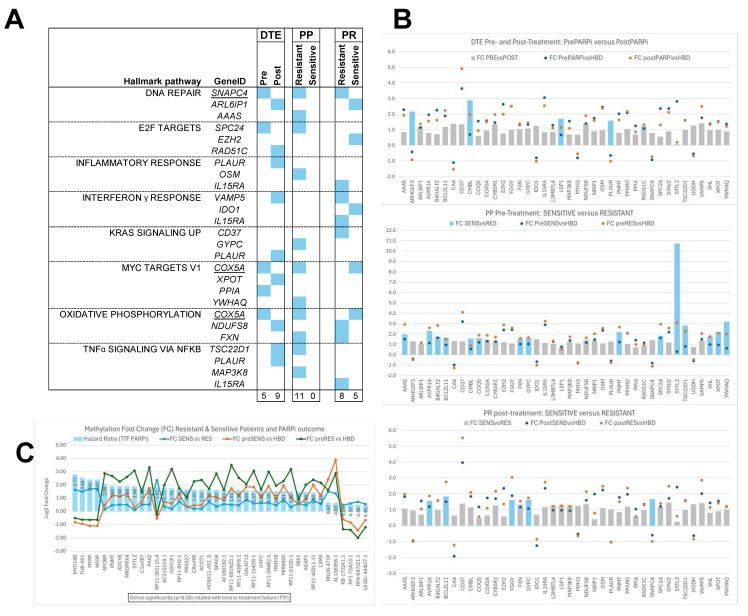
Cancer hallmark pathways (CHPs) and Epigenetic poly-ADP ribose-polymerase inhibitor (PARPi) hallmark differentially methylated regions (DMRs). Our epigenetic PARPi hallmark DMRs were evaluated specifically for CHPs, and these CHP-linked DMRs were further investigated for their methylation fold changes compared to healthy blood donors (HBDs) and between subsets. DMRs with fold changes below zero are hypomethylated and those with fold changes above zero are hypermethylated compared to HBDs. (**A**) Figure presenting the CHPs that were linked to at least 3 DMRs in one or more sample subsets, indicated by blue boxes. All CHPs were seen in all three epigenetic hallmarks, except the CHP interferon γ response, which was not observed in the PARPi predictive (PP) hallmarks. Moreover, the PP hallmarks only had CHP-linked DMRs in the resistant subset, while they have none in the sensitive subset. (**B**) Figure showing the fold changes observed for all CHP-linked DMRs per subset relative to HBDs (indicated by circles) and between subsets (shown as bars) for each epigenetic hallmark. The bars indicate DMRs with fold changes not significant (grey bars) or significant (blue bars) between subsets. (**C**) Figure presenting the 40 DMRs which were significantly associated with time to treatment failure (TTF) after PARPi therapy when evaluating the DMRs from the prePARPi subset using Cox proportional hazard logistic regression analyses. This figure shows the hazard ratio as blue bars, together with methylation fold changes relative to HBs (green/orange lines) and between resistant and sensitive patients (blue lines). Hypomethylated DMRs were associated most strongly with worse TTF (*MYO18B*) or with beneficial TTF (*LA16c-444G7.1*).

The epigenetic hallmark DMRs were also evaluated for CHPs (Figure 7). In total, 40 DMRs were linked to one or more of these CHPs. This analysis showed that at least three DMRs from the epigenetic hallmarks were linked either to DNA repair, *E2F* targets, inflammatory and interferon γ response, *KRAS* signaling and *MYC* targets, oxidative phosphorylation, or *TNFA NFKB* signaling (Figure 7A). Interestingly, the interferon γ response pathway was not seen in the PP hallmark, whereas all other pathways were only detected in the resistant samples of this hallmark. The CHP-linked DMRs were predominantly hypermethylated, i.e., fold changes above zero, in the different epigenetic hallmarks when compared to HBDs (Figure 7B). 

Significant fold changes (Blue bars in Figure 7B) were found between pre- and post-treatment samples (DTE: 5DMRs) and between sensitive and resistant patients in pre- (PP: 15DMRs) and post-treatment samples (PR: 5DMRs). Finally, another 40 DMRs from the epigenetic PP hallmarks that were significantly associated with TTF were investigated for their fold changes (Figure 7C). Remarkably, DMRs hypomethylated compared to HBDs were associated most strongly with worse TTF (*MYO18B*, *TUB-AS1*, and *MFRP*) or with beneficial TTF (*LA16c-444G7.1*, *RP4-647J21.1*, *RP1-72A23.1*, and *KB-1732A1.1*). 

## 4. Discussion

The examination of epigenetic and genomic characteristics in HGSOC patients undergoing PARP inhibitor maintenance therapy provides valuable insights into treatment response and disease progression. Our study delved into (dynamic) DNA genomic and methylation changes across disease conditions and treatment responses, revealing patterns indicative of the intricate regulatory mechanisms at play. Our simultaneous genome-wide genomic and epigenetic NGS analyses revealed that PARPi-resistant patients compared to HBDs and PARPi-sensitive and treatment-naïve EOC patients predominantly displayed cfDNA hypermethylation in pre-treatment blood, while aneuploid cfDNA and high tumor fractions were merely observed in post-treatment blood. 

The general workflow for our genome-wide cfDNA analyses started with the mFast-SeqS and MeD-seq analyses of all blood samples. These analyses were initially extended with shWGS combined with exome-seq only to samples with aneuploid cfDNA, but were ultimately completed in all post-treatment blood samples to reveal genomic differences at disease progression between PARPi-resistant and -sensitive patients, independent of tumor load. 

Our cheap and rapid mFast-SeqS analyses of both pre- and post-treatment blood showed only aneuploid cfDNA at disease progression from mainly PARPi-resistant patients. The lack of aneuploid cfDNA in pre-treatment blood was unexpected because cancer patients with late-stage disease often have high amounts of aneuploid cfDNA in their blood at baseline, as our previous studies in metastatic cancer patients have demonstrated [13,18,19]. However, the EOC patients in our PARPi cohort received (platinum-based) chemotherapy just before the start of PARPi maintenance therapy, which might explain the observed low tumor load in the pre-treatment blood. Moreover, the mFast-SeqS results at disease progression were independently confirmed by ichorCNA analyses of shWGS and exome-seq data, demonstrating higher tumor fractions in PARPi-resistant compared to PARPi-sensitive patients. 

Defining mutational signatures in cfDNA is challenging using shWGS with a low read depth coverage and due to there being much more germline DNA and lower tumor fractions in blood cfDNA compared to tumor tissue genomic DNA. Therefore, in our signature analyses, we used exome-seq with higher read depth coverages (median: 182 reads) and only selected variants which were not identified in the HBDs, were detected with at least 10 mutant reads, and with VAFs below 45% and between 55% and 70%, all to eliminate germline SNPs and to enrich for tumor-specific variants as much as possible. Subsequently, we examined signatures that were not identified in the HBDs to establish tumor biology-related signatures in the cancer blood samples. Using this approach, our analyses predominantly showed defective DNA mismatch repair- and chemotherapy-linked signatures in blood samples at disease progression. These mutational signatures make sense in our cohort of patients because they received platinum-based chemotherapy followed by PARPi maintenance therapy for their first recurrence. To summarize the genomic hallmarks, aneuploid cfDNA and high tumor fractions were seen in post-treatment blood mainly from PARPi-resistant patients, while the SBS26 signature for defective DNA mismatch repair was observed in PARPi-sensitive patients. These findings seem to be contrasting and unexpected, but can be explained due to the rapid disease progression in resistant patients, resulting in higher ctDNA loads. DNA mismatch repair has been described in ovarian cancer patients [20], but the association with PARPi therapy response, as seen in our samples, needs to be explored further in future studies. 

A comprehensive analysis was performed on MeD-seq data to provide detailed insights into methylation dynamics across different contexts, shedding light on disease progression, therapy response, and predictive markers [21]. For this, all cancer blood and tissue samples were compared to blood from HBDs. We observed that tissue samples exhibited a more balanced distribution of hypermethylation and hypomethylation among DMRs, in contrast with the consistent hypermethylation observed in blood samples across different time points. These observations emphasize the importance of considering tissue heterogeneity in epigenomic research. Interestingly, pre- and post-resistant PARPi samples predominantly displayed hypermethylated DMRs, suggesting a potential association between hypermethylation and disease progression and/or drug resistance. Further investigations into the molecular mechanisms underlying this association are warranted. 

Our research extensively explored methylation profiles across three distinct subclasses of sample subsets and defined different epigenetic PARPi hallmarks. Unique DMRs in pre- and post-treatment samples represented longitudinal changes in methylation due to disease progression and PARPi response. Therefore, these DMRs were defined as epigenetic DTE hallmarks. Next, DMRs found before disease progression and PARPi treatment in pre-treatment samples only seen in resistant or in sensitive patients were defined as epigenetic PARPi predictive (PP) hallmarks. Finally, DMRs found after disease progression and PARPi treatment in post-treatment samples of either resistant or sensitive patients were defined as epigenetic PARPi response (PR) hallmarks. Thus, these epigenetic hallmarks represent different stages of disease progression during PARPi maintenance therapy, i.e., before (PP), during (DTE), and after (PR) progression and/or treatment. Further (functional) studies are needed to establish which hallmarks and DMRs are associated with treatment and which are associated with disease progression. 

The identified epigenetic PARPi hallmarks contained hundreds of DMRs; however, only a few DMRs were reported as tumor driver genes (*SOX2*, *POU2AF1*, *PTK6*, *VHL*, *JAK3*, and *EZH2*) or as HRD genes (*RAD51C*). These genes have already been extensively investigated and linked to (ovarian) tumor progression, and some also to resistance for chemotherapy and PARPi. In particular, *RAD51C* methylation has been reported to be associated with PARPi resistance in HGSOC patients [22] and in cell line models [23]. Although we expected more tumor driver and HRD genes in the hallmarks, such as *BRCA1/2*, we will further investigate the other genes for their role in PARPi resistance. 

Our pathway analyses of the epigenetic hallmarks indicated that immune-related processes might be involved in PARPi therapy resistance. The identified transcription factor targets and CHPs might further help to pinpoint specific DMRs that play a role in PARPi response. We already identified several DMRs with significantly altered methylation levels between subsets from the different epigenetic hallmarks or that were associated strongly with TTF after PARPi treatment. Further research will be focused on these DMRs by validating our findings and evaluating in greater detail the most interesting DMRs for their relationship with disease progression and PARPi resistance in clinical samples but also in cell line models. 

## 5. Conclusions

Our genome-wide genomic and epigenetic analyses of blood cfDNA from HGSOC patients treated with PARPi maintenance therapy has shown that genomic hallmarks are mainly seen after treatment, whereas epigenetic hallmarks are present before and after treatment. Future studies are needed to validate the genomic findings but particularly the epigenetic hallmarks in pretreatment samples as putative predictive biomarkers for PARPi resistance.

## Figures and Tables

**Table 1:** Clinicopathological characteristics of poly-ADP ribose-polymerase inhibitor (PARPi) patient cohort.

PARPi Maintenance	Total	PARPi Resistant	PARPi Sensitive	*p*-Value *
**Total number of patients**	31	15	16	
**Age (median, range in years)**	68 [48–80]	70 [59–78]	65 [48–80]	0.186 *
**FIGO stage:**				
IIIB/IIIC	21	9	12	0.372
IV	10	6	4	
**Cytoreductive Surgery:**				
Primary debulking	5	2	3	0.358
Interval debulking	25	12	13	
No	1	1	0	
Surgical outcome **:				
Complete	22	10	12	0.843
Incomplete:	6	3	3	
<1 cm (optimal)	4	1	3	
>1 cm or irresectable	2	2	0	
Unknown	2	1	1	
**Previous lines of chemotherapy:**				
1–2	28	14	14	0.583
3–4	3	1	2	
**Platin-based chemotherapy:**				
Duration (median, range)	5 [2–7]	5 [4–7]	5 [2–6]	0.234 *
**PARPi type:**				
Olaparib	15	6	9	0.191
Niraparib	16	9	7	
**PARPi response:**				
Duration (median, range)	6 [2–43]	4 [2–6]	11 [6–43]	<0.001 *
≤6 months	15	15	0	
>6 months	16	0	16	
**BRCA1/2 status:**				
Mutated	9	3	6	0.181
Wid-type/unknown:	22	12	10	
Germline & tumor wild-type	11	6	5	
Germline wild-type	10	5	5	
Unknown	1	1	0	

* *p*-values are based on Student *t*-test; without * *p*-values are based on Χ^2^-test). ** Surgical outcome based on residual tumor.

## Data Availability

The data presented in this study are available upon request from the corresponding author, due to specified restrictions intended to safeguard the privacy of research participants.

## Supplementary Materials

The following supporting information can be downloaded at: https://www.mdpi.com/article/10.3390/genes15060750/s1. Figure S1: mFast-SeqS, shWGS and exome-seq analyses; Table S1: Patient BRCA1/2-status and genome-wide NGS findings.
