# Supplementary material for: Epigenetic and Genomic Hallmarks of PARP-Inhibitor Resistance in Ovarian Cancer Patients
Source: Genes (Basel). 2024 Jun 7;15(6):750. doi: 10.3390/genes15060750 (PMC11203368; doi:10.3390/genes15060750)
Supplement: Supplementary file 1 [file genes-15-00750-s001.zip › genes-3006722-supplementary.pdf]

genes

MDPI

Supplemental Table S1 – patient *BRCA1/2*-status and genome-wide NGS findings

Patient info		PARPi response		GWZ-score		Tumor Fraction		Identified SBS-signatures								
study ID	BRCA1/2 status	Type	duration (months)	Pre	Post	Exome	WGS	SBS3	SBS14	SBS17b	SBS25	SBS26	SBS35	SBS44	SBS84	SBS90
146	GT-wildtype	Resistant	2.2	2.1	45.4	0.24	0.26	0	0	0	0	0	0.0109	0	0.0250	0
223	G-wildtype	Resistant	3.2	1.6	2.7	0.06	0.04	0	0	0.0008	0	0	0	0	0.0247	0
355	G-wildtype	Resistant	3.4	2.1	2.5	0.08	0.08	0	0	0	0	0	0	0	0	0
247	GT-wildtype	Resistant	3.5	2.6	2.8	0.03	0.04	0	0	0.0014	0	0	0	0.0273	0.0175	0
231	G-wildtype	Resistant	3.6	2.9	5.1	0.11	0.07	0	0	0	0.0001	0	0	0	0.0198	0
199	unknown	Resistant	3.8	3.3	2.2	0.09	0.07	0	0	0	0	0	0	0	0	0
178	GT-wildtype	Resistant	4.1	1.6	69.3	0.30	0.30	0.0348	0	0.0036	0	0	0	0.0531	0.0313	0
300	mutation	Resistant	5.2	2.1	1.5	0.04	0.08	0	0	0	0	0	0	0	0.0649	0
115	GT-wildtype	Resistant	5.2	2.8	14.4	0.21	0.22	0	0	0	0	0	0	0	0.0148	0
59	mutation	Resistant	5.2	3.6	4.5	0.09	0.04	0	0	0	0	0	0	0	0.0157	0
134	GT-wildtype	Resistant	5.2	2.6	3.2	0.03	0.11	0.0349	0.0012	0.0011	0	0	0	0	0.0189	0
297	mutation	Resistant	5.3	2.5	2.9	0.11	0.12	0	0	0.0001	0	0	0	0	0	0
331	GT-wildtype	Resistant	5.7	3.9	4.1	0.15	0.03	0	0	0	0	0	0	0	0	0.0028
283	G-wildtype	Resistant	5.9	2.3	2.5	0.03	0.04	0	0	0	0	0	0	0	0	0
304	G-wildtype	Sensitive	6.5	2.0	2.7	0.04	0.04	0	0	0	0	0	0	0	0	0
354	G-wildtype	Sensitive	6.9	2.2	3.2	0.07	0.04	0	0	0.0086	0.0175	0.0461	0	0	0	0
316	GT-wildtype	Sensitive	7.7	3.3	3.4	0.08	0.04	0	0	0.0004	0	0	0	0	0.0207	0
391	GT-wildtype	Sensitive	8.1	3.5	1.8	0.11	0.05	0	0	0.0008	0	0	0	0	0.0283	0
215	G-wildtype	Sensitive	8.5	2.8	3.1	0.04	0.04	0	0	0.0002	0	0	0	0.0001	0.0179	0
177	GT-wildtype	Sensitive	10.0	2.7	2.0	0.09	0.06	0	0	0	0	0.0128	0	0	0.0214	0
282	G-wildtype	Sensitive	10.8	1.3	2.6	0.05	0.08	0	0	0.0023	0	0	0	0	0	0
181	GT-wildtype	Sensitive	10.9	2.6	3.8	0.09	0.06	0	0	0.0020	0	0	0	0	0.0338	0
295	G-wildtype	Sensitive	11.5	1.9	2.0	0.12	0.12	0	0	0	0	0	0	0	0	0
336	GT-wildtype	Sensitive	11.6	2.5	5.1	0.14	0.13	0	0	0	0	0.0022	0	0	0.0121	0
256	mutation	Sensitive	14.8	2.0	1.5	0.05	0.04	0	0	0	0	0.0187	0	0	0	0
294	mutation	Sensitive	16.7	2.7	3.5	0.05	0.05	0	0	0.0023	0	0	0	0	0.0200	0
72	mutation	Sensitive	21.3	2.0	3.4	0.13	0.08	0	0	0	0.0024	0	0	0	0.0322	0
170	mutation	Sensitive	23.2	1.7	3.2	0.03	0.04	0	0	0	0	0	0	0	0	0
226	mutation	Sensitive	23.2	1.6	1.9	0.03	0.05	0	0	0.0001	0	0	0	0	0	0
36	mutation	Sensitive	41.5	2.3	0.7	0.11	0.06	0	0	0	0	0	0	0	0	0

The BRCA1/2 status was determined in germline and for a subset of cases also in tumor tissue DNA. The status was indicated as mutated, or wildtype for germline only (G-wildtype) only for germline and tumor (GT-wildtype).

Supplemental Figure S1 – mFastSeqS, shWGS and exome-seq analyses

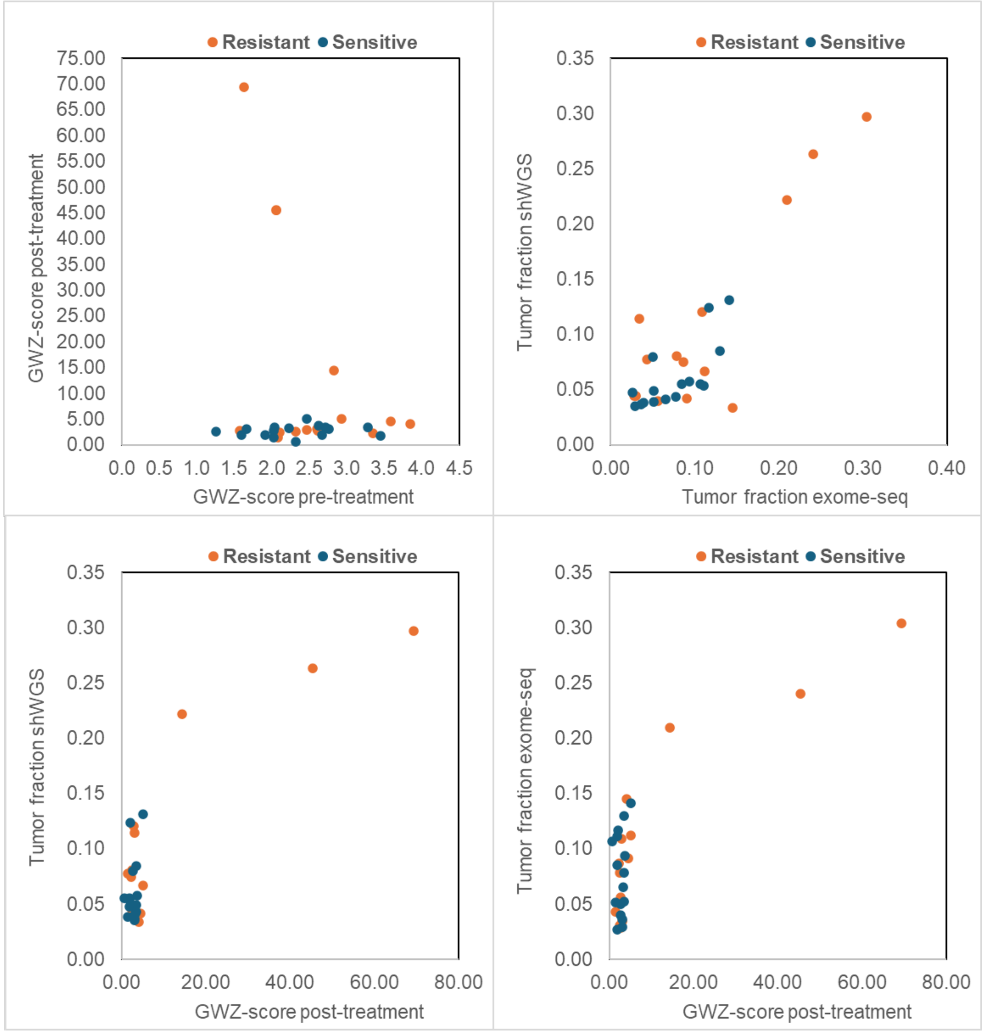

Blood taken before start of PARPi and after PARPi treatment were evaluated by mFastSeqS and shWGS and exome-seq for both PARPi-resistant and PARPi-sensitive patients. Genome-wide Z-scores were defined by mFastSeqS analyses, while tumor fractions were determined by ichorCNA analyses using shWGS and exome-seq data.
